# Neuroprotective and neurotoxic outcomes of androgens and estrogens in an oxidative stress environment

**DOI:** 10.1186/s13293-020-0283-1

**Published:** 2020-03-29

**Authors:** Phong Duong, Mavis A. A. Tenkorang, Jenny Trieu, Clayton McCuiston, Nataliya Rybalchenko, Rebecca L. Cunningham

**Affiliations:** 1 Department of Physiology and Anatomy, Institute for Healthy Aging, University of North Texas Health Science Center, Fort Worth, TX 76107 USA; 2 Texas College of Osteopathic Medicine, University of North Texas Health Science Center, Fort Worth, TX 76107 USA; 3 Department of Pharmaceutical Sciences, UNT System College of Pharmacy, University of North Texas Health Science Center, 3400 Camp Bowie Boulevard, Fort Worth, TX 76107 USA

**Keywords:** Neuroprotection, Neurodegeneration, Membrane androgen receptor, AR45, Window of opportunity, Estrogen receptors, Oxidative stress, Human hippocampus, Estrogen, Testosterone, Sex differences

## Abstract

**Background:**

The role of sex hormones on cellular function is unclear. Studies show androgens and estrogens are protective in the CNS, whereas other studies found no effects or damaging effects. Furthermore, sex differences have been observed in multiple oxidative stress-associated CNS disorders, such as Alzheimer’s disease, depression, and Parkinson’s disease. The goal of this study is to examine the relationship between sex hormones (i.e., androgens and estrogens) and oxidative stress on cell viability.

**Methods:**

N27 and PC12 neuronal and C6 glial phenotypic cell lines were used. N27 cells are female rat derived, whereas PC12 cells and C6 cells are male rat derived. These cells express estrogen receptors and the membrane-associated androgen receptor variant, AR45, but not the full-length androgen receptor. N27, PC12, and C6 cells were exposed to sex hormones either before or after an oxidative stressor to examine neuroprotective and neurotoxic properties, respectively. Estrogen receptor and androgen receptor inhibitors were used to determine the mechanisms mediating hormone-oxidative stress interactions on cell viability. Since the presence of AR45 in the human brain tissue was unknown, we examined the postmortem brain tissue from men and women for AR45 protein expression.

**Results:**

Neither androgens nor estrogens were protective against subsequent oxidative stress insults in glial cells. However, these hormones exhibited neuroprotective properties in neuronal N27 and PC12 cells via the estrogen receptor. Interestingly, a window of opportunity exists for sex hormone neuroprotection, wherein temporary hormone deprivation blocked neuroprotection by sex hormones. However, if sex hormones are applied following an oxidative stressor, they exacerbated oxidative stress-induced cell loss in neuronal and glial cells.

**Conclusions:**

Sex hormone action on cell viability is dependent on the cellular environment. In healthy neuronal cells, sex hormones are protective against oxidative stress insults via the estrogen receptor, regardless of sex chromosome complement (XX, XY). However, in unhealthy (e.g., high oxidative stress) cells, sex hormones exacerbated oxidative stress-induced cell loss, regardless of cell type or sex chromosome complement. The non-genomic AR45 receptor, which is present in humans, mediated androgen’s damaging effects, but it is unknown which receptor mediated estrogen’s damaging effects. These differential effects of sex hormones that are dependent on the cellular environment, receptor profile, and cell type may mediate the observed sex differences in oxidative stress-associated CNS disorders.

## Background

Sex differences have been of interest as far back as 1871 with Charles Darwin’s publication titled “The descent of man, and selection in relation to sex” [1]. Unfortunately, the number of studies on sex differences is sparse; leading to the National Institutes of Health (NIH) 2015 requirement of sex to be examined in NIH-funded studies. Further knowledge of sex differences is necessary as medicine is moving toward individualized precision medicine [2].

Numerous CNS disorders exhibit sex differences, which may result in the need for sex-specific standard of care. Men have an increased risk for Parkinson’s disease [3], autism [4], and schizophrenia [5]. Conversely, Alzheimer’s disease [6], major depression [7, 8], and stress disorders [9, 10] are more prevalent in women than men. Menopause in women also influences the prevalence of CNS disorders, such as Alzheimer’s disease [11], stroke [12], Parkinson’s disease [13–15], depression [16], anxiety disorders [17], and schizophrenia [18, 19]. During menopause, estradiol levels abruptly decline from 15–350 pg/ml (depending on menstrual cycle stage) to less than 10 pg/ml, which is below circulating estradiol levels in men (10–40 pg/ml) [20]. Interestingly, testosterone levels (8–60 ng/dL) are maintained in women during menopause [21–23]; these testosterone levels are 27–30 fold less than the levels observed in healthy young adult men (240–950 ng/dL) and middle-aged men (219–929 ng/dL) [24, 25].

Both androgens (i.e., testosterone and dihydrotestosterone, DHT) and estrogens (i.e., 17β-estradiol) along with their cognate receptors can influence the CNS by altering both structure and function [26, 27]. Within the past 25 years, studies have examined sex differences in androgen and estrogen receptor expression in the brain, such as the hypothalamus, cortex, and hippocampus (Table 1). Generally, sex hormones have protective effects in the CNS [36, 37]. However, recent findings indicate that the effects of sex hormones may depend on the cell type, cellular environment, and receptor expression profile [38–41]. Further, most studies only examined full-length androgen receptors, using antibodies (i.e., PG-21, Santa Cruz N-20, and Chemicon Ab561) that target amino acid sequences in the N terminus region of the androgen receptor (Table 1). These antibodies are unable to indicate the presence of an androgen receptor variant (i.e., AR45) that is missing the regulatory N terminus domain [42].

**Table 1:** Sex differences in steroid hormone receptor expression profile in the CNS

Receptor	Structure	Female: male expression	Citation
Androgen receptor—N terminal domain			
	Bed nucleus stria terminalis (adult, rat)	Male	[28]
	Cortex (PD 28 and adult, mice)	No difference	[29, 30]
	Hippocampus (PD 28, mice)	Male	[29]
	Hypothalamus (adult, mice)	Male	[30]
	Lateral septum (adult, mice)	Male	[31]
	Mammillary nucleus (adult, human)	Male	[32]
	Medial preoptic area (adult, mice)	Male	[31]
	Periventricular nucleus (adult, rat)	Male	[28]
	Spinal motor neurons (adult, rat)	No difference	[33]
	Substantia nigra pars Compacta (PD 30, rat)	No difference	[34]
	Substantia nigra pars reticulata (PD 30, rat)	Female	[34]
Estrogen receptor α			
	Cortex (PD 28, mice)	Male	[29]
	Hippocampus (PD 28, mice)	No difference	[29]
	Hypothalamus (adult, mice)	Female	[35]
	Substantia nigra pars compacta (PD 30, rat)	No difference	[34]
	Substantia nigra pars reticulata (PD 30, rat)	Female	[34]
Estrogen receptor β			
	Cortex (PD 28, mice)	Female	[29]
	Hippocampus (PD 28, mice)	Female	[29]
	Substantia nigra pars compacta (PD 30, rat)	Female	[34]
	Substantia nigra pars reticulata (PD 30, rat)	No difference	[34]

**Fig. 1:**
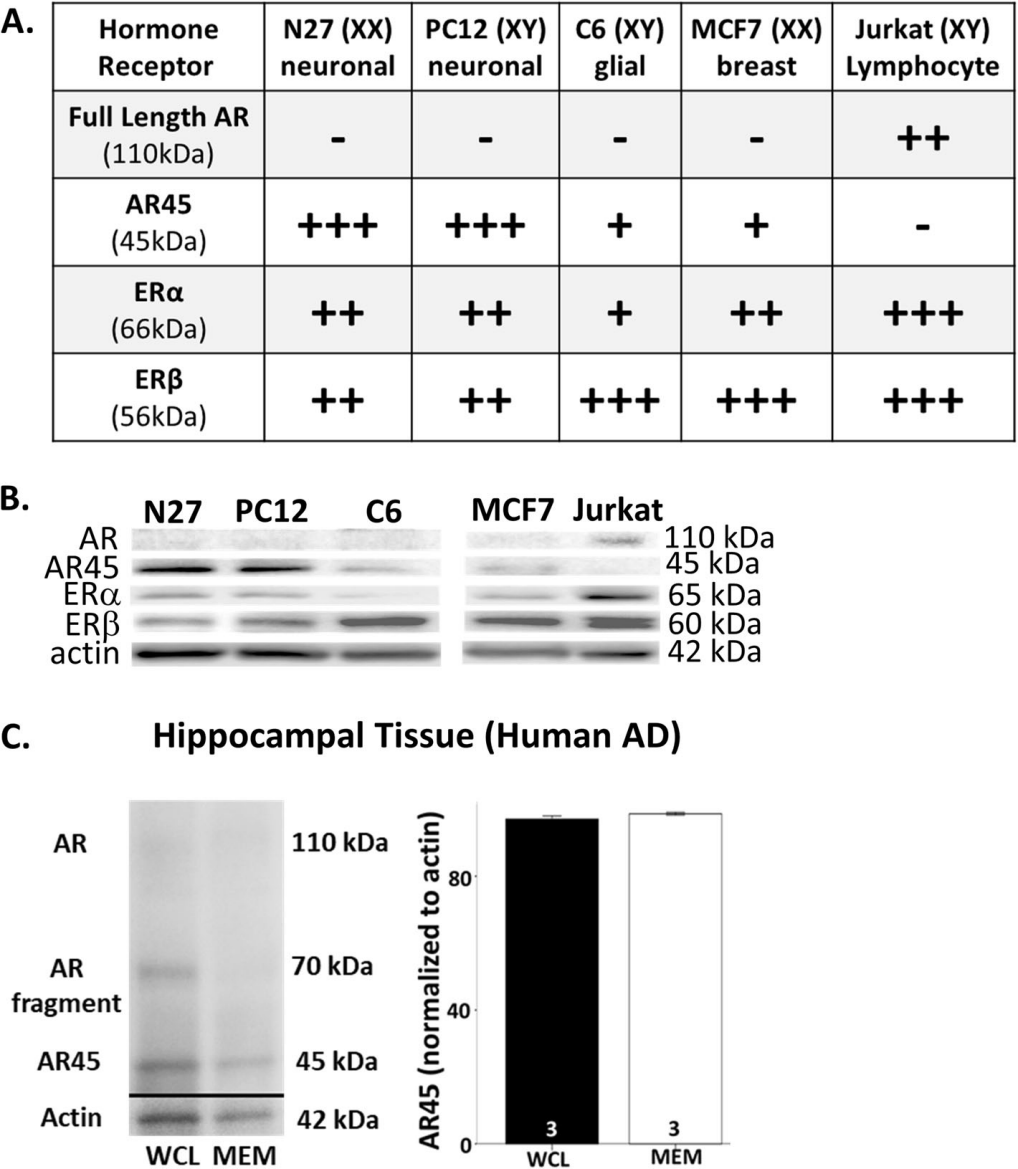
Expression profile of hormone receptors. N27, PC12, C6, and MCF7 cells express the androgen receptor splice variant, AR45, and not full-length androgen receptors. In contrast, Jurkat cells express the full-length androgens receptor but not the AR45, which indicate that Jurkat cells are useful as both positive control and negative control for full-length androgen receptors and AR45, respectively (**a**, **b**). AR45 protein is expressed in human hippocampal tissue (**c**). Using the C terminus domain androgen receptor antibody, AR-C19, AR45 expression was detected in the membrane fraction and the whole cell lysate of frozen hippocampal postmortem tissue obtained from donors diagnosed with Alzheimer’s disease. No significant differences were observed between AR45 expression in whole cell lysate versus the membrane fraction, indicating AR45 is a membrane-associated androgen receptor. As expected, full-length androgen receptors (110 kDa) were not observed in frozen samples. However, an androgen receptor fragment (70 kDa) was observed in whole cell lysate, indicating the full-length androgen receptor that resides in the cytosol and nucleus was degraded to fragments. To confirm AR45 protein expression, which is missing the N terminus domain (NTD), the AR-N20 NTD targeted androgen receptor antibody was used. No AR45 expression was detected with AR-N20 antibody. Results were determined by ANOVA. Results are reported as mean + SEM. *p* < 0.05; WCL, whole cell lysate; MEM, membrane fraction; AR, androgen receptor

The AR45 localizes to plasma membrane lipid rafts in multiple brain regions, such as the entorhinal cortex, the hippocampus, and the substantia nigra [42], and is present in the human postmortem brain tissue (Fig. 1c). This androgen receptor variant is unresponsive to classical androgen receptor antagonists and involved in non-genomic actions of androgens [43]. Specifically, AR45 interacts with G proteins and the NADPH oxidase (NOX) signaling pathways, which can lead to increased oxidative stress and cell loss [39, 42].

Oxidative stress results from the dysregulation of free radical homeostasis, which can damage lipids, proteins, and DNA. Free radicals are molecules that contain unpaired electrons and play important roles in cellular function (e.g., signal transduction and gene transcription) [43]. The most common free radicals are hydroxyls, superoxides, and nitric oxide, which can produce hydrogen peroxide and peroxynitrate. Further, the most common reactive oxygen species (ROS) that produce free radicals are hydrogen peroxide and peroxynitrate. These free radicals and ROS are primarily generated via mitochondrial aerobic metabolism to create energy (ATP production) [44].

The brain is the highest consumer of energy in the body, in which it uses 20% of available energy for cellular communication and housekeeping functions [45]. Under normal physiological conditions, ~ 2% of oxygen used to generate ATP is converted to ROS. In unhealthy or aged brains, more oxygen is converted to ROS [46], increasing the susceptibility of the brain to oxidative stress and damage. Oxidative damage can result in chronic diseases and has been shown to be associated with CNS diseases, such as Parkinson’s disease [47], autism [48, 49], schizophrenia [50], Alzheimer’s disease [51], stroke [52], major depression [53, 54], and anxiety disorders [55, 56]. Since sex differences are observed in these oxidative stress-associated CNS disorders and it is unclear what impact androgens and estrogens have on oxidative stress signaling, it is important to examine the relationship between sex hormones and oxidative stress.

In these studies, we focused on N27 neuronal-derived female rat cells, PC12 neuronal phenotype male rat cells, and C6 glial-derived male rat cells. These cell lines express estrogen receptors α/β and AR45, but do not express the full-length androgen receptor (Fig. 1a, b). These cell lines will allow further investigation of the effects of the novel androgen receptor variant, AR45, on cell survival. We chose to examine AR45 over the full-length androgen receptor as our prior studies found no effects of androgen receptor antagonists on oxidative stress endpoints, such as cell viability [39, 41].

## Materials and methods

### Reagents

Fetal bovine serum (FBS, 35-010-CV), Dulbecco’s modified Eagle’s medium (DMEM, 10-017-CV), and l-glutamine (25-005-CI) were purchased from Corning. Charcoal/dextran-stripped fetal bovine serum (CS-FBS, S11650) was purchased from Atlanta Biologicals. DMSO (D128), SuperSignal West Femto Substrate (34096), Pierce BCA Protein Assay Kit (23225), tris-buffered saline (TBS, BP2471), and Tween-20 (BP337) were purchased from Thermo Fisher Scientific. Penicillin-streptomycin solution (PS, 15140-122), phosphate-buffered saline (PBS, 10010-031), and TrypLE Select LE 10X (A12177-01) were purchased from Gibco. Androgen receptor degrader ASC-J9 (J9, HY-15194) was purchased from MedChem Express. ICI 182,780 (ICI, 1047) and Androgen R/NR3C4 (MAB5876) were purchased from R&D Systems. Actin (ADI-CSA-400) was purchased from Enzo Life Sciences. *tert-*Butyl hydroperoxide (H_2_O_2_, A13926) and thiazolyl blue tetrazolium bromide solution (MTT, L11939) were purchased from Alfa Aesar. RPMI-1640 medium (SH30027.02) and RPMI-1640 Phenol Red and l-glutamine-free medium (SH30605.01) were purchased from Hyclone. 4-Androsten-17β-ol-3-one (testosterone, A6950-000) and dihydrotestosterone 3-CMO: BSA (DHT-BSA, A2574-050) were purchased from Steraloids. 17β-Estradiol (E-8875) and NGF (N0513) were purchased from Millipore Sigma. The antibodies AR-C19 (SC-815), AR-N20 (SC-816), ERα (SC-542), and ERβ (SC-8974) were purchased from Santa Cruz Biotechnology. GAPDH (GTX627408) was purchased from GeneTex, and goat anti-rabbit (31460) and goat anti-mouse (31430) secondary antibodies were purchased from Invitrogen. NP40 lysis buffer (J619-500) was purchased from Ameresco. RIPA lysis buffer (N653) was purchased from VWR. 15-well/15 uL any KD mini protean gels (456-9036) and Immun-Blot PVDF membranes (162-0177) were purchased from Bio-Rad.

### Human cases

Frozen hippocampal postmortem tissue from male and female donors, aged 66–93, Caucasian, was obtained from the Institute for Healthy Aging’s Brain Bank at the University of North Texas Health Science Center. Tissue storage period was less than 9 years. All cases exhibited Alzheimer’s disease-associated pathology.

### Cell culture

This study used only rat-based cell lines in our experimental procedures. 1RB3AN27 (N27) dopaminergic cells (kind gift from Randy Strong, Ph.D., at University of Texas Health Science Center; RRID: CVCL_D584), PC12 dopaminergic adherent cells (ATCC CRL-1721.1; RRID: CVCL_F659), and C6 glial cells (ATCC CCL-107; RRID: CVCL_0194) were used in experimental paradigms. N27 cells are derived from SV40 T antigen-transformed neurons originally from embryonic female rat mesencephalic cells [57]. PC12 neuroblastic cells are derived from a pheochromocytoma from a male rat [58, 59]. C6 glioma cells were derived from the male rats [60, 61]. N27 and PC12 cells were grown in RPMI-1640 media supplemented with 10% FBS and 1% PS (culture media). Although the adherent PC12 cells do not require NGF differentiation for adherence, dopaminergic neuronal phenotype, and response to oxidative stressors [62], we added NGF (100 ng/ml) to the PC12 media at time of plating [63]. C6 cells were originally propagated in DMEM supplemented with 10% FBS and 1% PS. Following 48 h of growth, C6 cells were switched to the RPMI culture media. There were no significant differences observed in function or morphology following the medium switch in C6 cells (unpublished observation). Cells were maintained in a sterile environment at 37 ^o^C with 5% CO_2_ and sub-cultured every 2–3 days.

To ensure the quality and integrity of the different cell lines, all experiments were conducted between passages 16–21 (undifferentiated N27), 8–14 (undifferentiated C6), and 5–10 (differentiated PC12). We also characterized these cells based on their morphology, doubling time, and a well-characterized response to tert-butyl hydrogen peroxide (H_2_O_2_) and testosterone [39–41]. Cell lines were switched to RPMI 1640 serum-free media supplemented with 10% CS-FBS and 1% PS (experimental media) prior to induction of experimental compounds to avoid hormonal content found within regular FBS [39–41]. CS-FBS does not contain steroid hormones (i.e., estradiol, testosterone, thyroid hormones). Testosterone, estradiol, and DHT-BSA were from stock solutions made in DMSO (final DMSO concentration < 0.001%).

### Cell culture treatments

Reported LC-MS/MS brain hormone levels in male rats are (1) 5–24 nM testosterone [64–67], (2) 2.3–3.2 nM DHT [64, 67], and 0.2–0.9 nM estradiol [64, 67]. Since little to no albumin is present in healthy brains [68, 69], brain hormones are not protein bound and considered free. In order to compensate for the albumin (2.1 g/dl) in the CS-FBS, we used the Vermeulen calculation to determine the appropriate hormone dosage to attain physiological brain hormone levels [70, 71]. Therefore, in CS-FBS 100 nM, testosterone is 8 nM calculated free testosterone, 1 nM estradiol is 0.07 nM calculated free estradiol, and 500 nM DHT-BSA is 24 nM calculated free DHT-BSA. The higher DHT-BSA dosage was used to compensate for decreased hormone binding due to the 20 DHT molecules per 1 BSA molecule composition of DHT-BSA [39]. Based on this, the levels of hormones used in this study are a reasonable approximation of brain hormone levels.

N27 and C6 cells were plated onto 96-well plates at a density of 1.5–2.0 × 10^6^ cells/mL with culture media, whereas PC12 cells were plated at 6.0 × 10^4^ cells/mL in culture media. All cells were left to proliferate overnight, except for PC12 cells that required 48 h. For treatments under the neuroprotective paradigm, cells were exposed to testosterone [72], 17β-estradiol, and DHT-BSA in experimental media (i.e., CS-FBS) for 2 h. Following the hormone exposure, N27 cells were treated with 15–20 uM of H_2_O_2_ in experimental media, PC12 cells with 30 uM of H_2_O_2_ in experimental media, and C6 cells with 50 uM of H_2_O_2_ in experimental media for 18 h to induce 20–30% cell loss. Cells under the neurotoxic paradigm underwent 10–20 uM (N27), 30 uM (PC12), or 50 uM (C6) of H_2_O_2_ for 2 h to induce 20–30% cell loss before hormone exposure for an additional 18 h. Cell viability was determined following each treatment paradigm.

### Hormone receptor inhibitors

The estrogen receptor α/β inhibitor, ICI 182, 780 (ICI), and the androgen receptor degrader, ASC-J9 (J9), were used for this study. Inhibitor dose was chosen based on the IC_50_ data. Inhibitors and the degrader were made from stock solutions in DMSO (final DMSO concentration < 0.001%). For paradigms involving hormone receptor inhibitors, cells were exposed to ICI (300 pM) in experimental media for 1 h prior to induction of any hormones or oxidative stressor. For the androgen receptor degrader, J9 (5 uM), cells were exposed to the degrader for 30 min followed by either hormones or oxidative stressor for 1 h. Cells were then treated with either hormones or oxidative stressor for an additional 2 h.

### Cell viability

Cell viability was determined by MTT assay. Media was aspirated from all wells, replenished with 100 uL of RPMI-1640 phenol red-free medium, and supplemented with 10% CS-FBS, 1% PS, and 1% l-glutamine. This was followed by the addition of 20 uL of 5 mg/mL of MTT solution to each well. Experimental plates were then covered in foil to block additional light and incubated at 37 ^o^C with 5% CO_2_ for 3 h. Following incubation, plates were read at an absorbance of 595 nm. The colorimetric intensity is directly proportional to the number of viable cells in each well. Readings from respective treatment groups were then normalized to the vehicle control group to determine cell viability [39–41]. Three independent experiments, using different cell cultures, were conducted.

### Western blot analysis

In order to confirm hormone receptor expression (Fig. 1), untreated N27, PC12, and C6 cells were collected and homogenized. We included Jurkat whole cell lysate (Abcam ab7899) as a positive control for the full-length androgen receptor and estrogen receptors α/β, and the MCF7 breast cancer cell line (ATCC HTB-22; RRID: CVCL_0031) was used as a positive control for estrogen receptors α/β. Additionally, we examined androgen receptor expression in human hippocampal tissues (25–50 ug). Tissues were homogenized using a RIPA lysis buffer mixture supplemented with protease inhibitor cocktail, 1 uM DTT, and 1 mM EDTA. Following homogenization and separation into whole cell lysate and membrane fractions [42], protein concentrations were determined using the Pierce BCA Protein Assay Kit per manufacturer’s instructions. Equal amounts of protein (20 ug) were separated in a Bio-Rad Any KD polyacrylamide gel at 25 mA for approximately 1 h and transferred onto a PVDF membrane at 50 V at 4 ^o^C for 2–3 h. Following transfer, membrane blots were blocked using 5% non-fat milk in TBST for 30 min at room temperature. After blocking, membranes were incubated with constant agitation in primary antibodies (ERα 1:1000, ERβ 1:1000, ARC19 1:1000 for cell lines, Androgen R/NR3C4 1:1000 and ARN20 1:1000 for human tissue, Actin 1:1000, and GAPDH 1:10000) in 1% TBST non-fat milk either 2 h at room temperature or overnight at 4 ^o^C. Membranes were then washed with 10% TBST twice for 10 min each before being incubated with secondary antibodies (Goat Anti-Rb HRP 1:1000 and Goat Anti-Ms HRP 1:10000) in 1% TBST non-fat milk for 30 min at room temperature. Afterwards, membranes were washed with 10% TBST two times for 10 min each. Visualization of bands was performed using SuperSignal West Femto Maximum Sensitivity Substrate and imaged for 30–90 s. Band intensity was then quantified by densitometry using the National Institutes of Health ImageJ program and normalized to GAPDH or actin levels. Three independent experiments were used.

### Statistical analysis

Analyses were performed using IBM SPSS Statistics version 21 software. Statistical comparisons were made by two or three-way ANOVA using oxidative stressor (H_2_O_2_), hormones (testosterone, 17β-estradiol, DHT-BSA), and inhibitors as independent factors. This was followed by Fisher’s LSD post hoc analysis to evaluate differences between groups. Results are expressed as mean± SEM, and *p* value less than or equal to 0.05 (*p*≤ 0.05) indicates statistically significant differences. Each experiment was replicated at least three times using different cell cultures.

## Results

### Testosterone and 17β-estradiol are protective in N27 and PC12 cells but not C6 cells

**Fig. 2:**
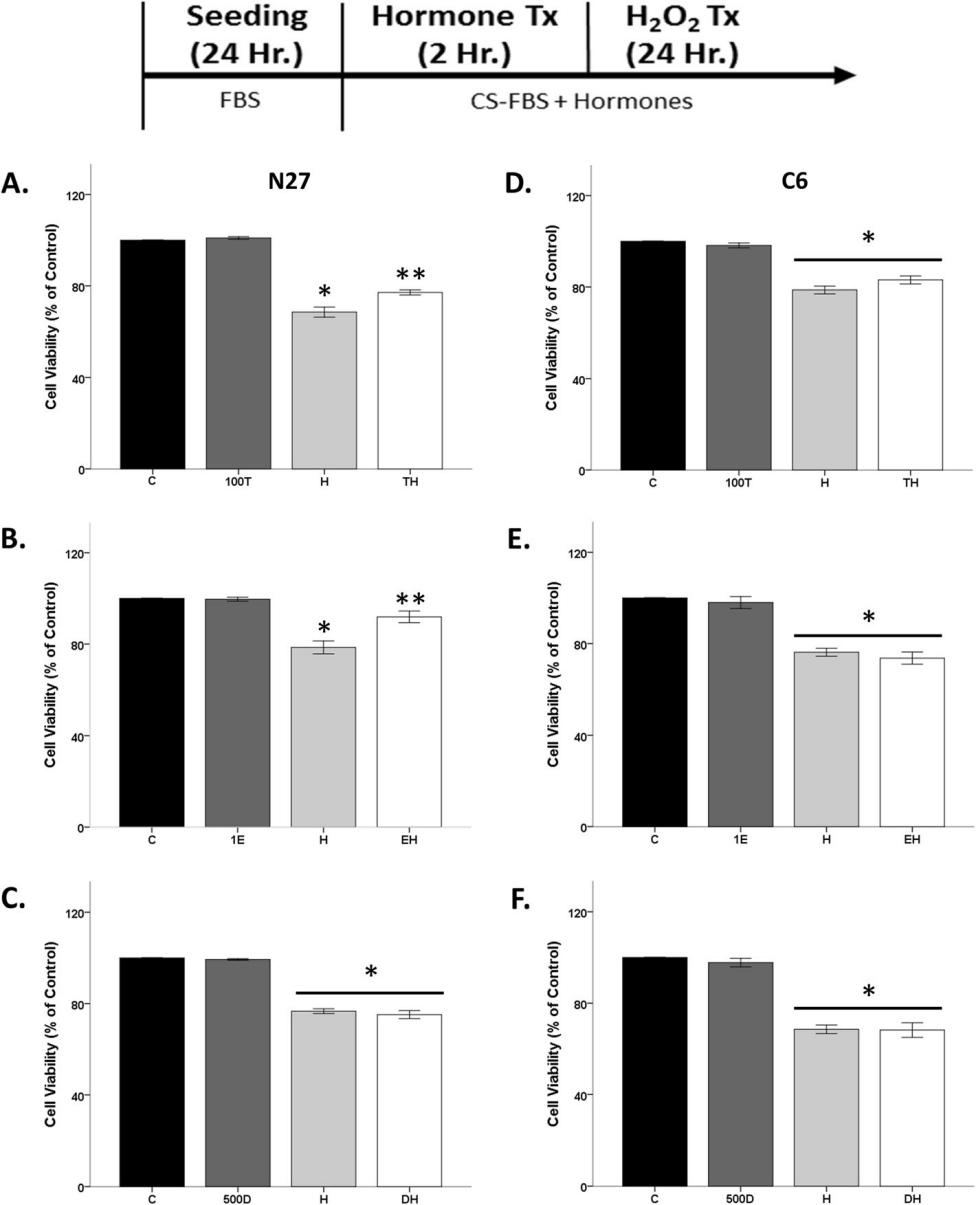
An effect of hormone pretreatment in N27 and C6 cells in the presence of oxidative stress. Hormone alone has no effect on cell viability. H_2_O_2_ decreased the cell viability by ~ 20–30%. Testosterone and 17β-estradiol pretreatment prior to H_2_O_2_ is protective in N27 cells (**a**, **b**) and not in C6 cells (**d**, **e**). DHT-BSA, followed by H_2_O_2_ exposure, did not protect N27 and C6 cells in an oxidative stress environment (**c**, **f**). Results were determined by ANOVA followed by Fisher LSD post hoc test. Results are reported as mean + SEM. *p* < 0.05; *versus control, **versus H_2_O_2_. C, vehicle control; T, 100 nM testosterone; H, H_2_O_2_; TH, pre-treatment T. E, 1 nM 17β-estradiol; EH, pre-treatment E; D, 500 nM DHT-BSA; DH, pre-treatment D

In vitro testosterone-mediated neuroprotection has been observed in several neuronal cells, including N27 cells [40, 73]. Similarly, in this study, we observed significant effects of the oxidative stressor H_2_O_2_ (*F*_1, 8_ = 475.2, *η*^2^ = 0.94, *p* < 0.05) and the hormone testosterone (*F*_1, 8_ = 13.8, *η*^2^ = 0.03, *p* < 0.05) on cell viability, along with an interaction between H_2_O_2_ and testosterone (*F*_1, 8_ = 8.7, *η*^2^ = 0.02, *p* < 0.05). Two-hour pretreatment of N27 cells with testosterone (100 nM) prior to oxidative stress, protected the cells by attenuating H_2_O_2_-induced cell loss (Fig. 2a). Consistent with our prior studies, testosterone alone did not have any effect on cell viability [39–41].

To examine if 17β-estradiol protects against oxidative stress damage, N27 cells were pretreated with 17β-estradiol, followed by H_2_O_2_. Significant effects of oxidative stressor H_2_O_2_ (*F*_1, 8_ = 56.2, *η*^2^ = 0.64, *p* < 0.05) and 17β-estradiol (*F*_1, 8_ = 11.2, *η*^2^ = 0.13, *p* < 0.05), along with an interaction between H_2_O_2_ and estradiol (*F*_1, 8_ = 12.5, *η*^2^ = 0.14, *p* < 0.05) on cell viability, were observed (Fig. 2b).

Unlike testosterone and 17β-estradiol, DHT-BSA did not alter H_2_O_2_-induced cell loss in N27 cells (Fig. 2c). DHT and 17β-estradiol are testosterone metabolites, which are ligands for the androgen receptor and the estrogen receptor, respectively. The addition of the BSA molecule to DHT restricts its activity to membrane androgen receptors. These results indicate that the putative membrane-associated androgen receptor does not mediate steroid hormone neuroprotection against oxidative stress insults, suggesting that the estrogen receptor mediates testosterone- and estrogen-mediated neuroprotection.

To determine if this effect is specific to neuronal-derived cells, we examined the neuroprotective properties of testosterone, 17β-estradiol, and DHT-BSA in C6 glial-derived cells. Similar to N27 cells, this cell line does not contain full-length androgen receptors but does express the androgen receptor variant, AR45 (Fig. 1). Unlike the N27 cells, neither testosterone (Fig. 2d) nor estradiol (Fig. 2e) was protective against H_2_O_2_-induced cell loss in C6 cells. Likewise, DHT-BSA did not protect the cells from H_2_O_2_-induced cell loss (Fig. 2f).

**Fig. 3:**
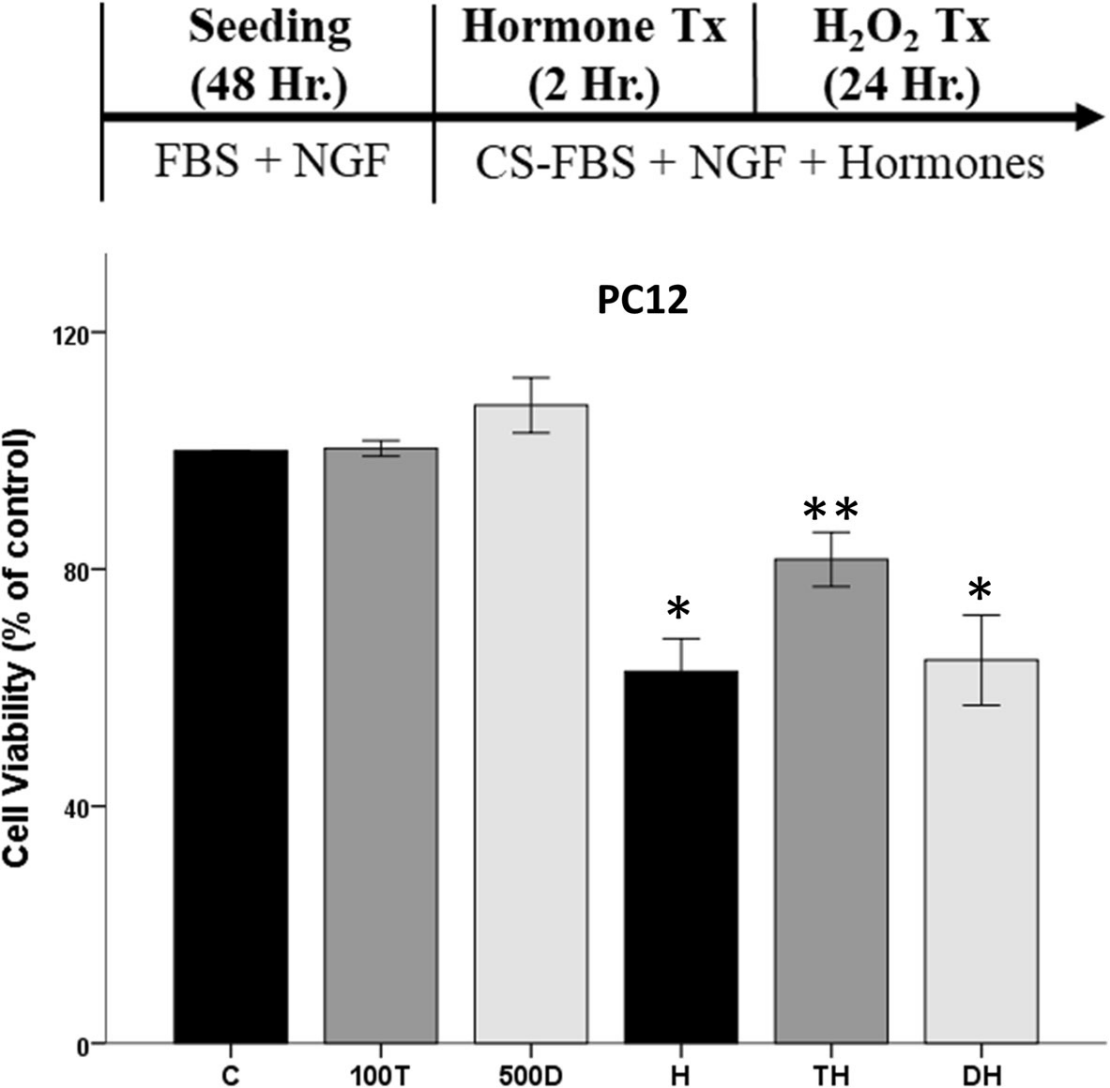
Effect of hormone pretreatment in PC12 cells in the presence of oxidative stress. Hormone alone has no effect on cell viability. H_2_O_2_ decreased cell viability by ~ 30%. Testosterone pretreatment prior to H_2_O_2_ is protective in PC12 cells. DHT-BSA, followed by H_2_O_2_ exposure, did not protect PC12 cells in an oxidative stress environment. Results were determined by ANOVA followed by Fisher LSD post hoc test. Results are reported as mean + SEM. *p* < 0.05; *versus control, **versus H_2_O_2_. C, vehicle control; T, 100 nM testosterone; H, H_2_O_2_; TH, pre-treatment T; D, 500 nM DHT-BSA; DH, pre-treatment D

Since the N27 cells are a female-derived cell line, we conducted similar experiments in the PC12 male-derived cell line to examine if sex chromosome complement may underlie this protective effect in neuronal-like cells (Fig. 3). We observed significant effects of the oxidative stressor H_2_O_2_ (*F*_1, 24_ = 73.64, *η*^2^ = 0.68, *p* < 0.05) on cell viability, along with an interaction between H_2_O_2_ and hormones (*F*_2,24_ = 3.418, *η*^2^ = 0.06, *p* < 0.05). Two-hour pretreatment of PC12 cells with testosterone (100 nM) prior to oxidative stress protected the cells by attenuating H_2_O_2_-induced cell loss. In contrast, DHT-BSA did not alter H_2_O_2_-induced cell loss in PC12 cells. These findings are similar to results in the female-derived N27 cells.

Interestingly, two different concentrations of H_2_O_2_ were necessary to induce 20% cell loss in the cell lines. The N27 and PC12 cells were more sensitive to H_2_O_2_ (20–30 uM) than C6 cells to H_2_O_2_ (50 uM). This sensitivity to H_2_O_2_ may be due to dopamine metabolism of N27 and PC12 cells, which increases the oxidative stress burden and possibly sensitizes the cells to subsequent oxidative stressors [40, 74].

### Estrogen receptor mediates neuroprotection

**Fig. 4:**
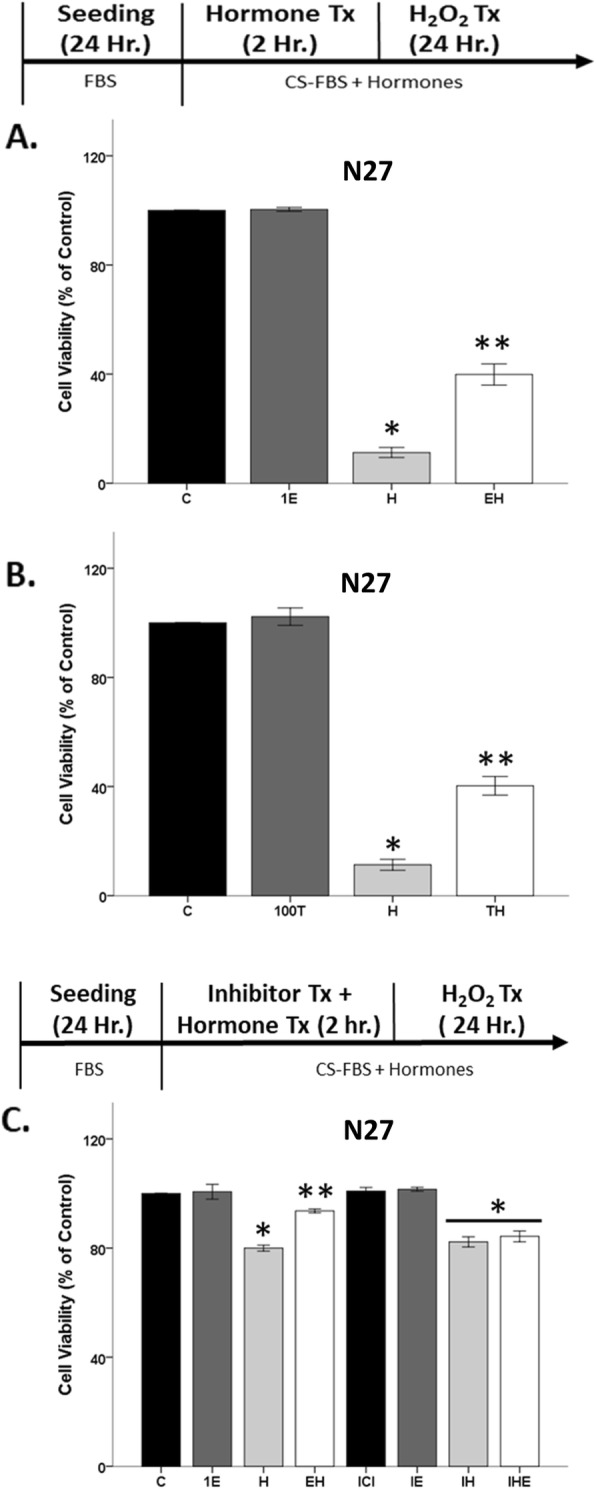
Neuroprotection is via estrogen receptor. 17β-estradiol and testosterone pretreatment prior to oxidative stressor is neuroprotective, regardless of oxidative stress insult (**a**, **b**). Estrogen receptor antagonist, ICI, was able to block 17β-estradiol’s neuroprotective action, but did not impact H_2_O_2_-induced cell loss (**c**). Results were determined by ANOVA followed by Fisher LSD post hoc test. Results are reported as mean + SEM. *p* < 0.05; *versus control, **versus H_2_O_2_. C, vehicle control; E, 1 nM 17β-estradiol; H, H_2_O_2_; EH, pre-treatment E; ICI, estrogen receptor antagonist. T, 100 nM testosterone; TH, pre-treatment T

Since protection was not observed in C6 cells, we focused on N27 cells for further investigation into the mechanisms underlying hormone-mediated protection. Both testosterone and estradiol were protective against a mild oxidative stress insult that caused 20% cell loss (Fig. 2a, b), and thus, we wanted to ensure that these hormones would be protective against harsher oxidative stress insults. Therefore, we increased the H_2_O_2_ concentration to 50 uM to induce 80% cell loss (Fig. 4a, b). 17β-estradiol pretreatment significantly protected the cells from oxidative stress-induced cell death (Fig. 4a), as evidenced by significant effects of H_2_O_2_ (*F*_1, 8_ = 1176.8, *η*^2^ = 0.93, *p* < 0.05), estradiol (*F*_1, 8_ = 44.3, *η*^2^ = 0.03, *p* < 0.05), and an interaction between H_2_O_2_ and estradiol (*F*_1, 8_ = 42.2, *η*^2^ = 0.03, *p* < 0.05) on cell viability. Similarly, testosterone pretreatment protected N27 cells from H_2_O_2_-induced cell loss (Fig. 4b), in which significant effects of H_2_O_2_ (*F*_1, 8_ = 877.7, *η*^2^ = 0.93, *p* < 0.05) and testosterone (*F*_1, 8_ = 37.7, *η*^2^ = 0.04, *p* < 0.05), along with an interaction between these two factors (*F*_1, 8_ = 27.5, *η*^2^ = 0.03, *p* < 0.05), were observed.

To determine if the estrogen receptor is mediating neuroprotection, we blocked the estrogen receptor with ICI that inhibits estrogen α/β receptors [75]. We observed significant effects of H_2_O_2_ (*F*_1, 16_ = 210.7, *η*^2^ = 0.86, *p* < 0.05), estradiol (*F*_1, 16_ = 15.3, *η*^2^ = 0.06, *p* < 0.05), and an interaction between H_2_O_2_ and estradiol (*F*_1, 16_ = 11, *η*^2^ = 0.04, *p* < 0.05) on cell viability (Fig. 4c). No effects of 17β-estradiol alone were observed. Approximately 20% cell loss was induced by H_2_O_2_. As expected, 17β-estradiol protected the cells from H_2_O_2_’s neurotoxic effects. This neuroprotective effect of estradiol on cell viability was blocked by the co-application of ICI (300 pM, IC50 concentration) with 17β-estradiol prior to H_2_O_2_ (*F*_1, 16_ = 7.2, *η*^2^ = 0.03, *p* < 0.05), confirming that 17β-estradiol’s neuroprotective effect is through the estrogen α/β receptor (Fig. 4c).

### Hormone neuroprotection lost when cells are temporarily hormone deficient

**Fig. 5:**
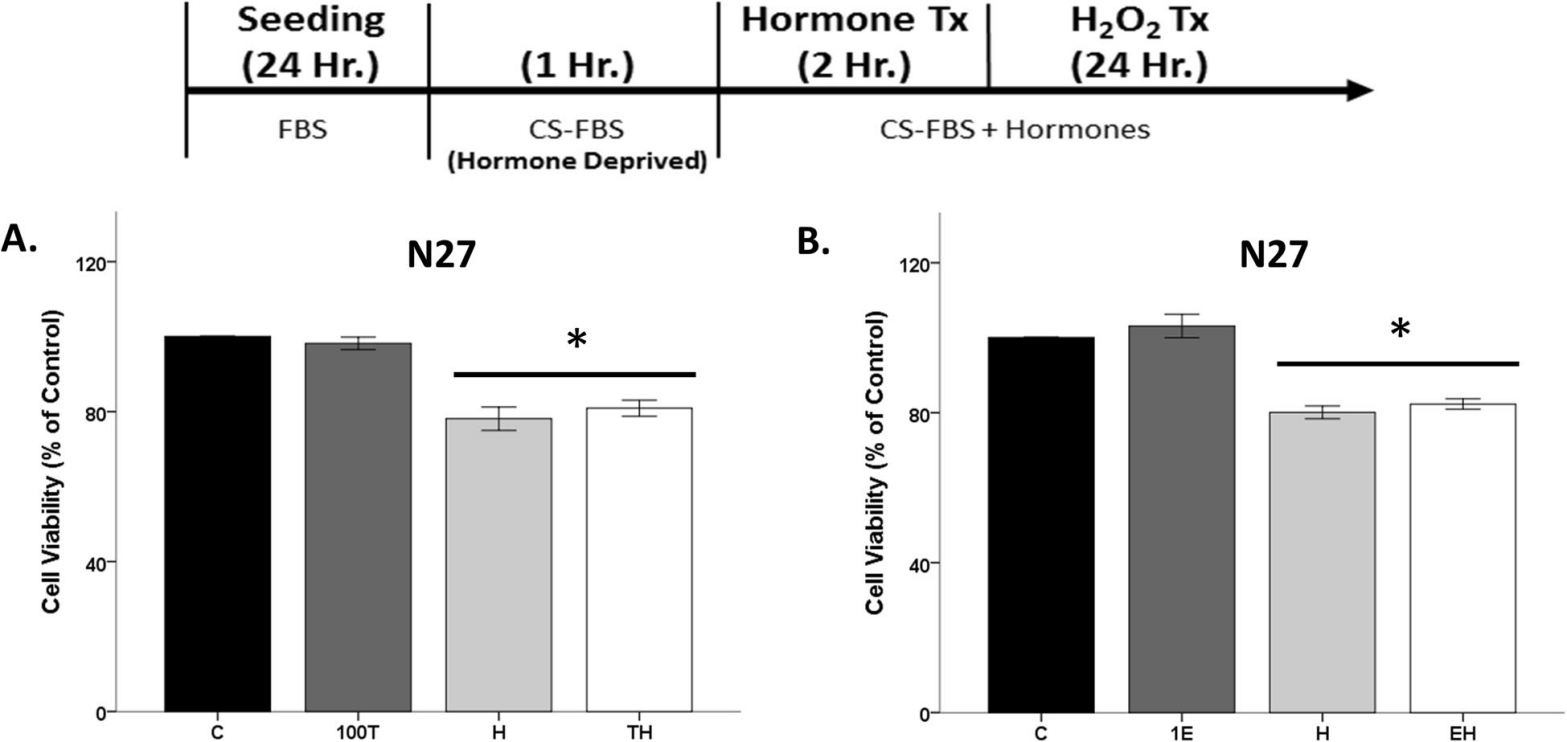
Neuroprotection lost after 1-h hormone deprivation. Testosterone and 17β-estradiol had no effect on cell viability, regardless of oxidative stress. H_2_O_2_ decreased cell viability by ~ 20% (**a**, **b**). Results were determined by ANOVA followed by Fisher LSD post hoc test. Results are reported as mean + SEM. *p* < 0.05; *versus control; C, vehicle control; E, 1 nM 17β-estradiol; H, H_2_O_2_; EH, pre-treatment E; T, 100 nM testosterone; H, H_2_O_2_; TH, pre-treatment T

Even though the FBS media (~ 27.5 pg/ml estradiol) was replaced with CS-FBS media in the prior experiments, cells were always exposed to hormones due to exogenous hormone application (e.g. testosterone, 17β-estradiol) in CS-FBS media. In this set of experiments, N27 cells were incubated in CS-FBS media in the absence of hormones 1 h prior to exogenous hormone (17β-estradiol and testosterone) treatment for 2 h (Fig. 5). After hormone treatment, cells were exposed to H_2_O_2_. Interestingly, neither 17β-estradiol nor testosterone-protected N27 cells from H_2_O_2_-induced cells loss (Fig. 5). These results highlight a “window of opportunity” for hormone action, in which hormone deprivation for at least 1 h was sufficient to ameliorate the neuroprotective effects of 17β-estradiol and testosterone against a subsequent oxidative stressor.

### Androgens and estrogens are damaging in an oxidative stress environment

**Fig. 6:**
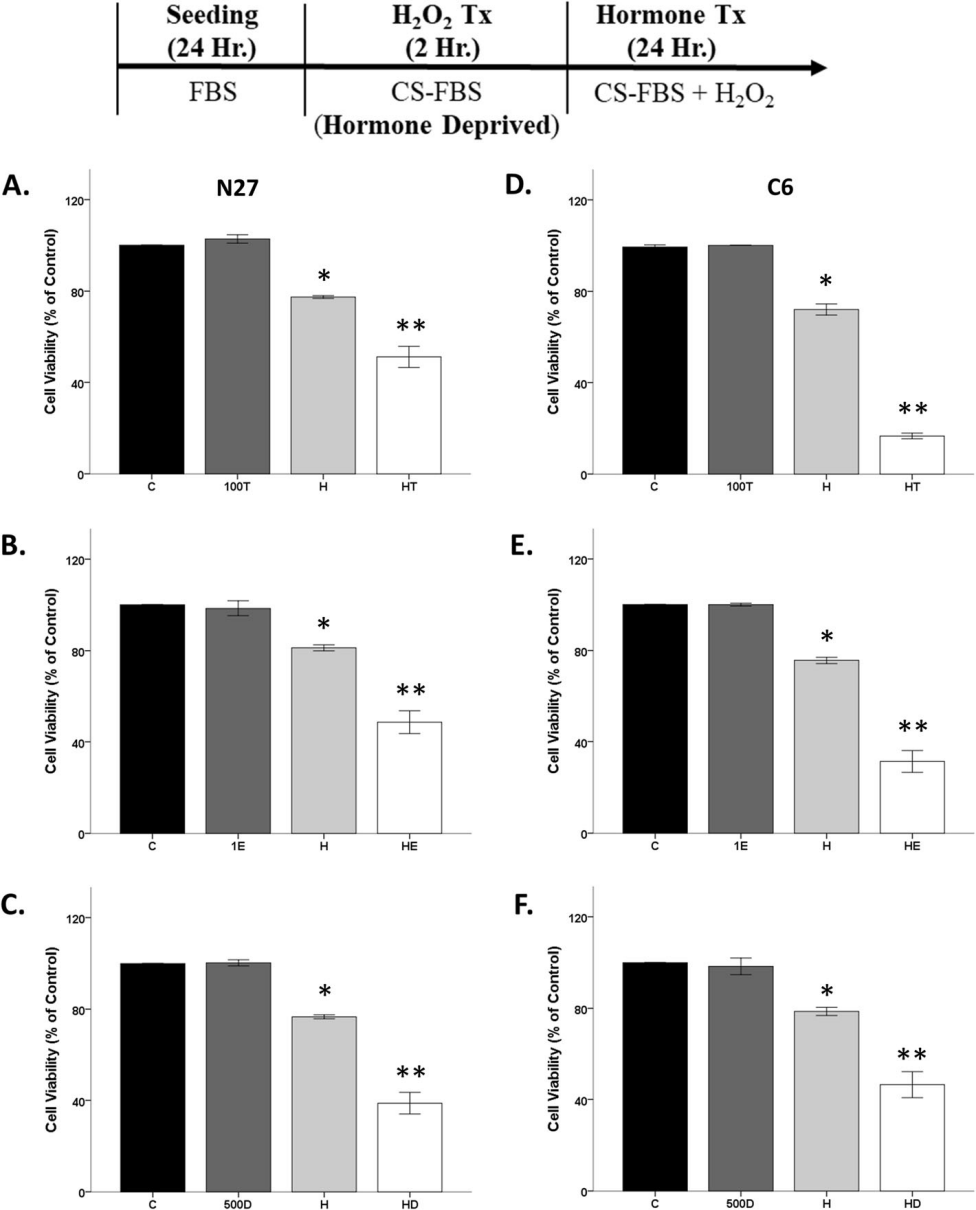
Neurotoxic effects of hormones in an oxidative stress environment. Alone, testosterone, 17β-estradiol, and DHT-BSA have no effect on cell viability. H_2_O_2_ decreased cell viability by ~ 20%. Testosterone, 17β-estradiol, and DHT-BSA further decreased cell viability in the presence of oxidative stress in N27 cells (**a**–**c**) and C6 cells (**d**–**f**). Results were determined by ANOVA followed by Fisher LSD post hoc test. Results are reported as mean + SEM. *p* < 0.05; *versus control, **versus H_2_O_2_. C, vehicle control; T, 100 nM testosterone; H, H_2_O_2_; HT, post-treatment T. E, 1 nM 17β-estradiol; HE, post-treatment E; D, 500 nM DHT-BSA; HD, post-treatment D

**Fig. 7:**
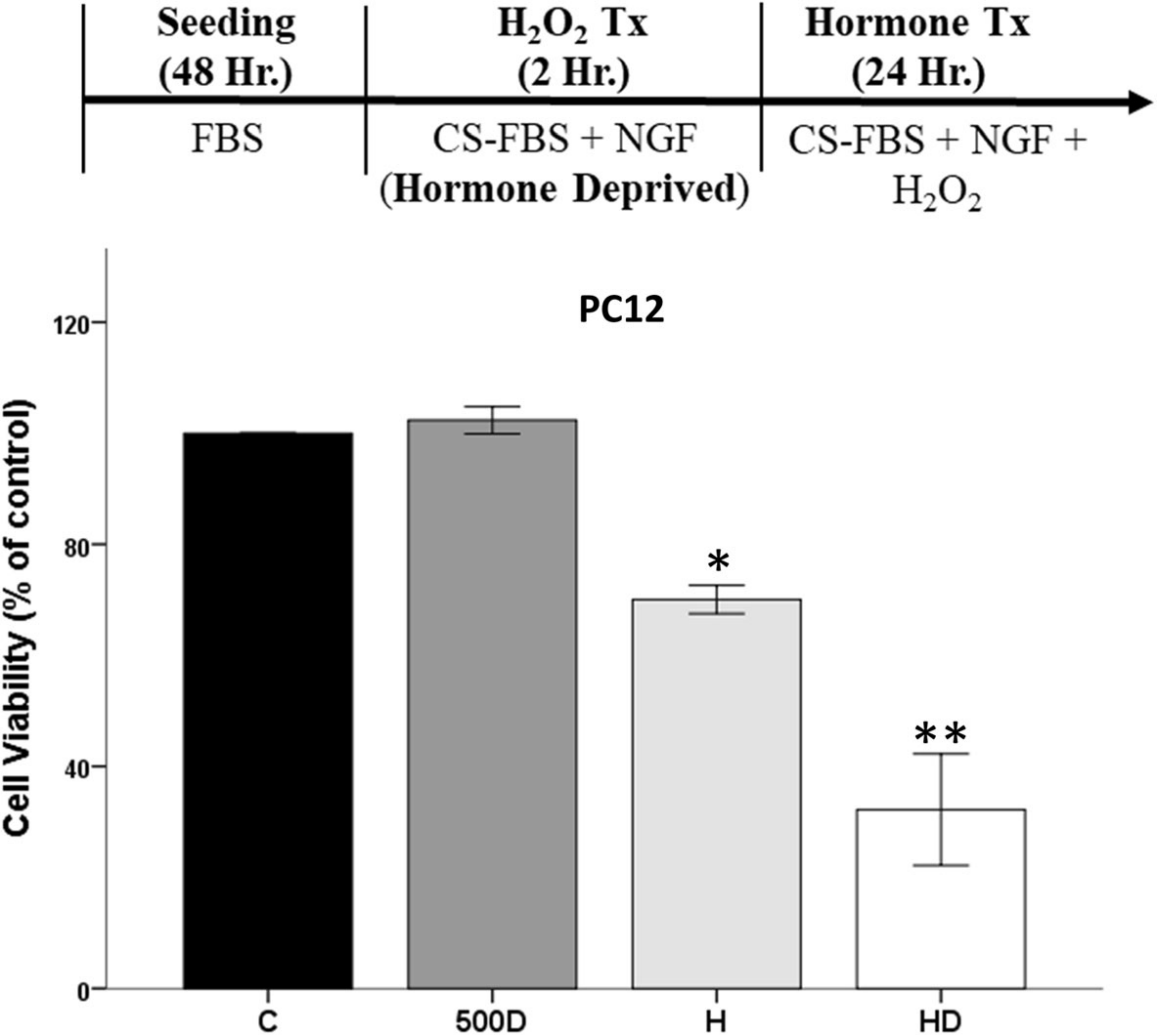
Neurotoxic effects of hormones in an oxidative stress environment on PC12 cells. Alone, DHT-BSA had no effect on cell viability. H_2_O_2_ decreased cell viability by ~ 30%. DHT-BSA further decreased cell viability in the presence of oxidative stress in PC12 cells. Results were determined by ANOVA followed by Fisher LSD post hoc test. Results are reported as mean + SEM. *p* < 0.05; *versus control, **versus H_2_O_2_. C, vehicle control; H, H_2_O_2_; D, 500 nM DHT-BSA; HD, post-treatment D

We previously published that testosterone in an oxidative stress environment is damaging in N27 cells [39–41]. In this study, testosterone, 17β-estradiol, and DHT-BSA further decreased cell viability in the presence of oxidative stress in both N27 and C6 cells (Fig. 6). Regardless of cell line, hormone alone did not have any effect on cell viability. In N27 cells, significant effects were observed with H_2_O_2_ (*F*_1, 8_ = 222.6, *η*^2^ = 0.78, *p* < 0.05), testosterone (*F*_1, 8_ = 22.4, *η*^2^ = 0.08, *p* < 0.05), and an interaction between H_2_O_2_ and testosterone (*F*_1, 8_ = 34, *η*^2^ = 0.12, *p* < 0.05) on cell viability (Fig. 6a). Estradiol had similar effects as testosterone on N27 cell viability in an oxidative stress environment (Fig. 6b), as evidenced by significant effects with H_2_O_2_ (*F*_1, 8_ = 129.4, *η*^2^ = 0.67, *p* < 0.05), 17β-estradiol (*F*_1, 8_ = 31.9, *η*^2^ = 0.16, *p* < 0.05), and an interaction between H_2_O_2_ and 17β-estradiol (*F*_1, 8_ = 26.8, *η*^2^ = 0.14, *p* < 0.05) on cell viability. Consistent with our prior studies [41], the membrane androgen receptor agonist, DHT-BSA, exacerbated H_2_O_2_-induced cell loss (Fig. 6c), as shown by significant effects with H_2_O_2_ (*F*_1, 8_ = 287.2, *η*^2^ = 0.70, *p* < 0.05), DHT-BSA (*F*_1, 8_ = 56.2, *η*^2^ = 0.14, *p* < 0.05), and an interaction between H_2_O_2_ and DHT-BSA (*F*_1, 8_ = 58.1, *η*^2^ = 0.14, *p* < 0.05). Similar to N27 cells, we observed damaging effects of hormones in an oxidative stress environment in PC12 cells. Significant effects were observed with H_2_O_2_ (*F*_1, 12_ = 88.064, *η*^2^ = 0.7, *p* < 0.05), DHT-BSA (*F*_1, 12_ = 11.027, *η*^2^ = 0.09, *p* < 0.05), and an interaction between H_2_O_2_ and DHT-BSA (*F*_1, 12_ = 14.198, *η*^2^ = 0.11, *p* < 0.05) on cell viability (Fig. 7).

Unlike our results that showed only neuroprotection in the N27 cell line, androgens and estrogens exacerbated H_2_O_2_-induced cell loss in the C6 cells. Specifically, testosterone (*F*_1, 8_ = 383.3, *η*^2^ = 0.17, *p* < 0.05), H_2_O_2_ (*F*_1, 8_ = 1488.2, *η*^2^ = 0.66, *p* < 0.05), and an interaction between H_2_O_2_ and testosterone (*F*_1, 8_ = 364.1, *η*^2^ = 0.16, *p* < 0.05) were observed in C6 cell viability (Fig. 6d). We observed the same responses with 17β-estradiol (*F*_1, 8_ = 77.7, *η*^2^ = 0.15, *p* < 0.05), H_2_O_2_ (*F*_1, 8_ = 341.9, *η*^2^ = 0.68, *p* < 0.05), and an interaction between the two variables (*F*_1, 8_ = 77.6, *η*^2^ = 0.15, *p* < 0.05) on cell viability (Fig. 6e). Figure 6f shows the same response with the membrane androgen receptor agonist, DHT-BSA, in which there were significant effects of H_2_O_2_ (*F*_1, 8_ = 109.2, *η*^2^ = 0.69, *p* < 0.05), DHT-BSA (*F*_1, 8_ = 23.2, *η*^2^ = 0.15, *p* < 0.05), and an interaction between oxidative stressor and DHT-BSA (*F*_1, 8_ = 19, *η*^2^ = 0.12, *p* < 0.05) on cell viability.

### Cytosolic estrogen and androgen receptors do not mediate hormone toxicity

**Fig. 8:**
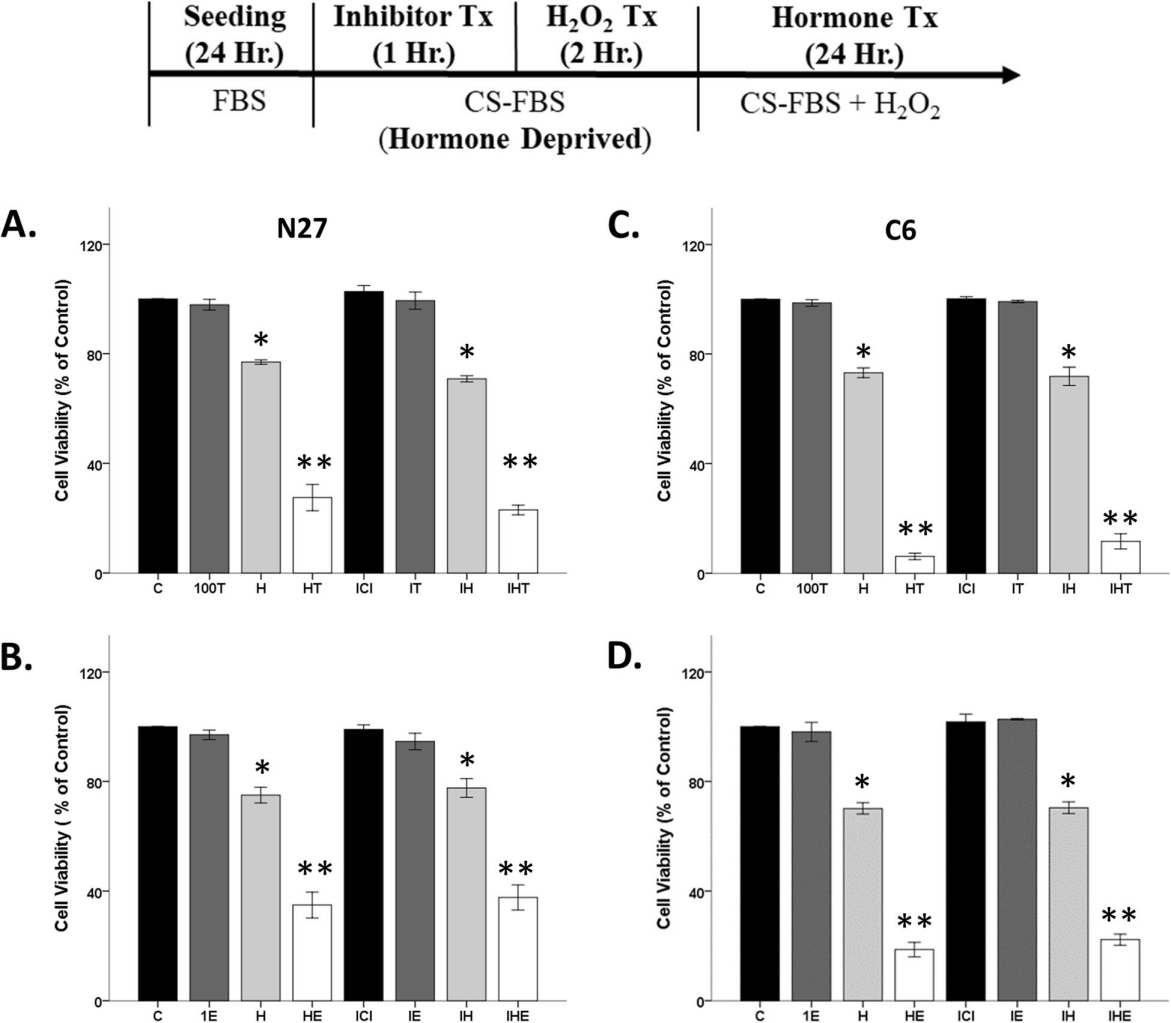
The role of estrogen receptor in hormone-induced neurodegeneration. Estrogen receptor antagonist, ICI, did not prevent testosterone and 17β-estradiol’s detrimental effects after H_2_O_2_ exposure in N27 cells (**a**, **b**) and C6 cells (**c**, **d**). Results were determined by ANOVA followed by Fisher LSD post hoc test. Results are reported as mean + SEM. *p* < 0.05; *versus control, **versus H_2_O_2_. C, vehicle control; T, 100 nM testosterone; H, H_2_O_2_; HT, post-treatment T. E, 1 nM 17β-estradiol; HE, post-treatment E

Our prior studies show that inhibiting cytosolic androgen receptors with flutamide, enzalutamide, or bicalutamide does not block androgen’s damaging effects in an oxidative stress environment [39, 40]. It is unknown what role estrogen receptors play in the damaging effects of testosterone or estradiol. Since estrogen α/β receptors are involved in neuroprotection (Fig. 4c), it is possible that these receptors may also mediate their damaging effects. Using N27 cells, ICI did not block testosterone’s negative effects in an oxidative stress environment (Fig. 8a), as evidence by significant effects of H_2_O_2_ (*F*_1, 16_ = 864.1, *η*^2^ = 0.67, *p* < 0.05), testosterone (*F*_1, 16_ = 224.4, *η*^2^ = 0.17, *p* < 0.05), and an interaction between oxidative stressor and testosterone (*F*_1, 16_ = 179.8, *η*^2^ = 0.14, *p* < 0.05) on cell viability but no effects of ICI (*F*_1, 16_ = 0.152, *η*^2^ = 0.0001, *p* > 0.05). A similar lack of response was observed with ICI and estradiol in an oxidative stress environment (Fig. 8b), wherein H_2_O_2_ (*F*_1, 16_ = 347, *η*^2^ = 0.66, *p* < 0.05), 17β-estradiol (*F*_1, 16_ = 96.7, *η*^2^ = 0.18, *p* < 0.05), and an interaction between H_2_O_2_ and 17β-estradiol (*F*_1, 16_ = 66.9, *η*^2^ = 0.13, *p* < 0.05) had significant effects on N27 cell viability but not ICI (*F*_1, 16_ = 0.030, *η*^2^ = 0.00006, *p* > 0.05).

Likewise, in C6 cells ICI did not block testosterone or estradiol exacerbation of H_2_O_2_-induced cell loss. In Fig. 8c, C6 cell viability was significantly impacted by H_2_O_2_ (*F*_1, 16_ = 2162.1, *η*^2^ = 0.63, *p* < 0.05), testosterone (*F*_1, 16_ = 657.5, *η*^2^ = 0.19, *p* < 0.05), and an interaction between oxidative stressor and testosterone (*F*_1, 16_ = 610.5, *η*^2^ = 0.18, *p* < 0.05), but no effects of ICI (*F*_1, 16_ = 1.589, *η*^2^ = 0.0005, *p* > 0.05). Similar effects were found using estradiol in Fig. 8d, in which we observed significant effects of H_2_O_2_ (*F*_1, 16_ = 1235.5, *η*^2^ = 0.70, *p* < 0.05), 17β-estradiol (*F*_1, 16_ = 256.1, *η*^2^ = 0.15, *p* < 0.05), and an interaction between oxidative stressor and 17β-estradiol (*F*_1, 16_ = 246.5, *η*^2^ = 0.14, *p* < 0.05), but no effects of ICI (*F*_1, 16_ = 0.006, *η*^2^ = 0.00, *p* > 0.05) in C6 cells.

### Non-genomic mechanisms underlie hormone toxicity

Since neither estrogen receptor α/β nor cytosolic androgen receptors mediate hormone toxicity, we focused on the AR45 androgen receptor variant that is expressed in plasma membrane lipid rafts in the CNS [42]. We previously published that ASC-J9 (J9), an androgen receptor degrader, was able to protect the N27 cells from testosterone’s damaging effects in an oxidative stress environment [39]. However, it is unknown if AR45 mediates androgen toxicity in C6 cells.

**Fig. 9:**
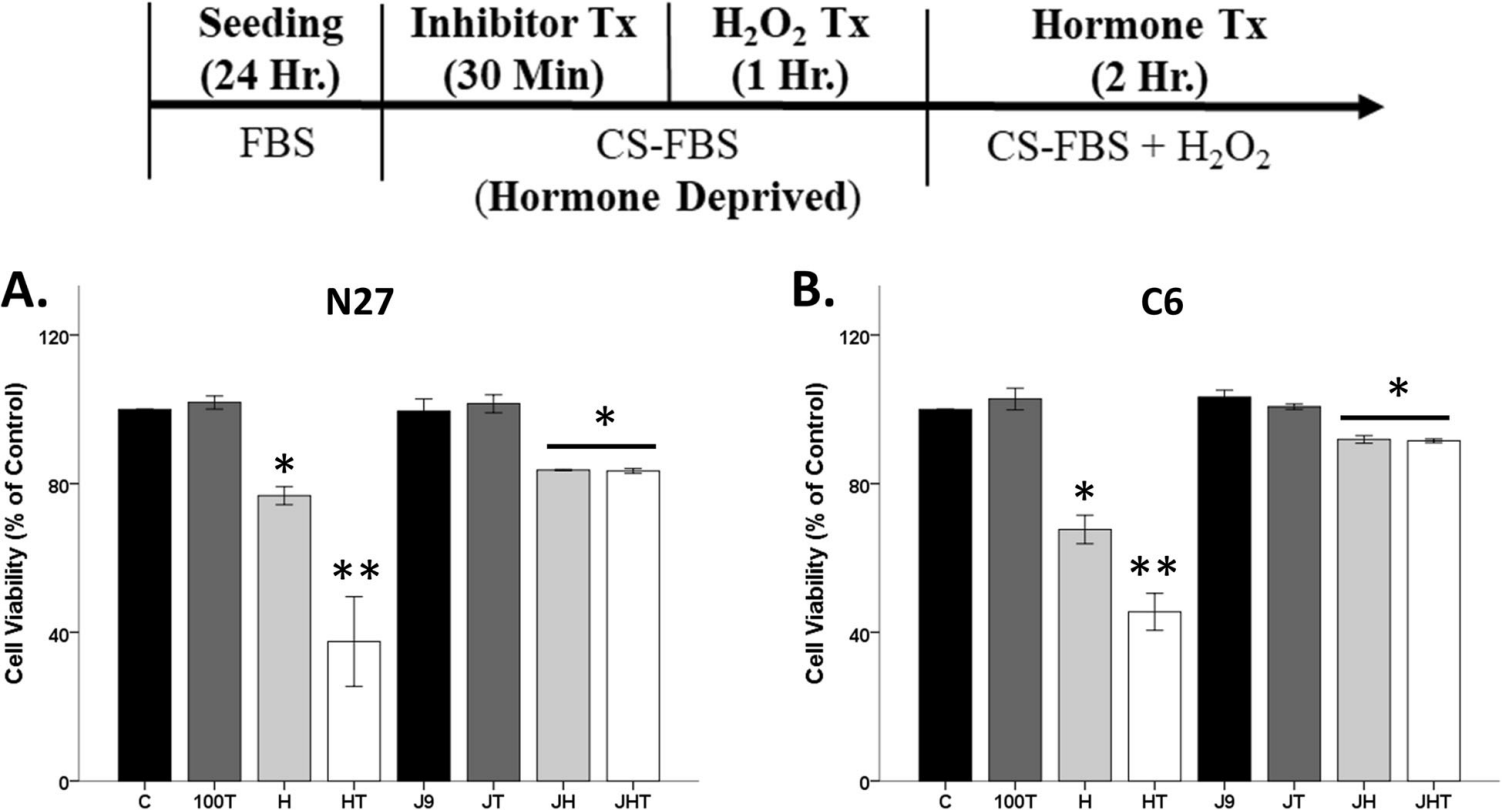
Androgen receptor degradation blocked testosterone-induced neurodegeneration in N27 cells. Cells were seeded for 24 h, followed by J9 pretreatment for 30 min. At 80% confluency, cells were exposed to H_2_O_2_ for 1 h followed by testosterone treatment for 2 h. J9 blocked further exacerbation of H_2_O_2_-induced cell loss in N27 cells (**a**). However, J9 attenuated H_2_O_2_-induced cell loss in C6 cells, and thus, its effect on testosterone could not be determined (**b**). Results were determined by ANOVA followed by Fisher LSD post hoc test. Results are reported as mean + SEM. *p* < 0.05; *versus control, **versus H_2_O_2_. C, vehicle control; H, H_2_O_2_; T, 100 nM testosterone; H, H_2_O_2_; HT, post-treatment T; J9, ASC J9

Similar to our prior studies, degradation of the AR45 via J9 protected N27 cells from testosterone-induced cell loss in the presence of H_2_O_2_ (Fig. 9a). We observed significant effects of H_2_O_2_ (*F*_1, 16_ = 86.2, *η*^2^ = 0.67, *p* < 0.05), testosterone (*F*_1, 16_ = 7.5, *η*^2^ = 0.06, *p* < 0.05), and a significant interaction between H_2_O_2_ and testosterone (*F*_1, 16_ = 10.9, *η*^2^ = 0.08, *p* < 0.05), along with a significant interaction between H_2_O_2_, testosterone, and AR degrader (*F*_1, 16_ = 8.8, *η*^2^ = 0.07, *p* < 0.05). Notably, J9 did not affect H_2_O_2_-induced cell loss, indicating that it does not have off-target scavenging effects at the current concentration in N27 cells.

We observed different results using C6 cells. Contrary to the observable neuroprotective actions by J9 in N27 cells, J9 acts as a scavenger in C6 cells. Specifically, J9 attenuated H_2_O_2_-induced cell loss (*F*_1, 16_ = 90, *η*^2^ = 0.25, *p* < 0.05), and thus, its effect on testosterone could not be determined (Fig. 9b). A lower dose (1 uM) of J9 was used. However, it was ineffective and did not impact C6 cell viability (data not shown).

## Discussion

The role of sex hormones, such as androgens and estrogens, on cellular function is unclear. For example, some studies show androgens and estrogens are protective in the CNS. However, other studies found either no effects or damaging effects, especially in oxidative stress environments. Interestingly, sex differences have been observed in multiple oxidative stress-associated CNS disorders, such as Parkinson’s disease [47], autism [48, 49], schizophrenia [50], Alzheimer’s disease [51], stroke [52], major depression [53, 54], and anxiety disorders [55, 56], indicating a role for sex hormones. Since it is unclear what impact androgens and estrogens have on oxidative stress signaling, the current study examined the relationship between sex hormones and oxidative stress on cell viability.

The major findings of this study are (1) the first evidence of AR45 protein expression in the human brain, (2) testosterone and estrogen are protective against subsequent oxidative stress insults in neuronal-derived cells but not in glial-derived cells, (3) the estrogen receptor α/β mediates sex hormone neuroprotection, (4) a 1-h window of opportunity exists for sex hormone neuroprotection, (5) sex hormone administration following oxidative stress exacerbates oxidative stress damage in neuronal- and glial-derived cells, (6) the estrogen receptor is not involved in sex hormone-mediated toxicity, and (7) AR45 mediates androgen exacerbation of oxidative stress-induced cell loss. Since no differences were observed due to sex chromosome complement (N27 female-derived and PC12 male-derived neuronal phenotypic cells, C6 male-derived glial cells), the observed findings indicate that sex hormones’ cellular effects are not dependent on genotype (XX, XY) but rather are more specific to cell type, receptor profile, and the environmental status of the cell (e.g., oxidative stress load).

Although it is known that the full-length androgen receptor is expressed in the hippocampus [76, 77], our data showed lack of full-length androgen receptor expression in frozen hippocampal postmortem tissue from individuals diagnosed with Alzheimer’s disease (Fig. 1c). This result is not unexpected, as the full-length androgen receptor protein is known to degrade into fragments (e.g., 70 kDa fragments) under conditions such as freezing [42, 78, 79]. Further, the presence of 70 kDa androgen receptor fragment increases with age [80]. Interestingly, aging is associated with increased oxidative stress [81, 82]. Thus, it is a possible oxidative stress may play a role in androgen receptor degradation, as oxidative stress has a bidirectional relationship with calcium-dependent calpain proteases [83–85] that can cleave full-length androgen receptors into 70 kDa fragments [86–89]. Future studies will examine if aging and neurodegenerative disorders are associated with increased expression of androgen receptor fragments.

The first study on the characterization and distribution of AR45 in humans by Ahrens-Fath 2005 failed to observe AR45 expression in the human brain tissue [90]. The Ahrens-Fath study used the whole brain tissue, whereas we used a specific region of the brain tissue (i.e., hippocampus). The Ahrens-Fath study failed to show full-length androgen receptor transcript expression (positive control) in the human brain [90], which is widely known to be present in the human brain [32, 77]. Therefore, the lack of full-length androgen receptor and AR45 transcript expression in the human brain in the Ahrens-Fath study could be a false negative result. Since our data shows that AR45 protein is present in the human brain tissue and does not respond to classical androgen receptor antagonists along with data from Hu [91] showing AR45 mRNA expression in the aged human brain tissue, this protein may be an important pharmacological therapeutic target for neurodegenerative conditions.

Neuronal- and glial-derived cells responded differently to the oxidative stressor, H_2_O_2_. The neuronal phenotypic N27 and PC12 cell lines were more sensitive to oxidative stress than the glial-derived C6 cells. This result is consistent with several reported studies [92–94]. Glia cell (e.g., astrocytes and microglia) functions are diverse. They range from maintaining the brain environment [95], energy storage, and synaptic maintenance by modulating the neurotransmitter release and uptake (e.g., glutamate and GABA) [96–101], regulating the action potentials via potassium modulation [102, 103], and synthesizing and releasing the neurotrophic factors and neurosteroids [104–107]. These glial cell functions may underlie their resistance to oxidative stress insults.

The role of glial cells in neuronal degeneration is of increasing interest. The glia to neuron ratio (GNR) may play a role in the observed sex differences in brain regions linked with oxidative stress-associated diseases. In this study, we reviewed the literature for GNR in various brain regions associated with Alzheimer’s disease, Parkinson’s disease, major depression, anxiety disorders, schizophrenia, and autism spectrum disorders, as these disorders exhibit sex differences in prevalence (Table 2). We generally observed fewer glial cells per neuron in brain regions linked with more male-biased oxidative stress CNS disorders. For example, in the striatum, substantia nigra pars compacta, spinal cord, and cerebellum, neuronal cells far outnumbered glial cells. The presence of fewer glial cells could increase the susceptibility of these brain regions to oxidative stress damage due to the loss of glial supportive mechanisms from oxidative stress damage. Interestingly, testosterone itself is an oxidative stressor [39–41, 129]. Our results show that under conditions of oxidative stress, testosterone can exacerbate oxidative stress damage via a membrane-associated androgen receptor (AR45) [39–41, 129]. Therefore, androgens could be involved in the observed sex differences in these brain regions.

**Table 2:** Glia to neuron ratio (GNR) in various brain regions associated with oxidative stress-related CNS disorders

Brain region	GNR	Citations for GNR	Associated disorders	Citations for disorders
Cortex (monkey)	1:1	[108]	Female bias: AD, MD, ANX Male bias: PD, SZ, ASD	[109–114]
Cortex (human)	4:1	[115]		
Cortex (rat)	1:1	[116]		
Striatum (mouse)	1:17	[117]	Female bias: MD	[110, 111, 113]
			Male bias: PD, SZ, ASD	
Basal ganglia (human)	1:2	[118]	Female bias: MD,	[110, 111, 113, 114]
			Male bias: PD, SZ, ASD	
Substantia nigra pars compacta (mouse)	1:9	[119]	Female bias:—	[110, 120]
			Male bias: PD, SZ	
Thalamus (human)	17:1	[121]	Female bias: MD	[111, 113, 122]
			Male bias: PD, SZ	
Hippocampus overall (mouse)	1:1	[117]	Female bias: AD, MD, ANX, Male bias: PD, SZ, ASD	[109–114]
CA1 (mouse)	1:2	[117]		
CA3 (mouse)	1:3	[117]		
Locus coeruleus (human)	27:1	[123]	Female bias: AD, MD	[110–112, 124, 125]
			Male bias: PD, ANX	
Amygdala basolateral (rat)	1:7	[117, 126]	Female bias: AD, MD, ANX,	[110, 112, 114, 127]
			Male bias: PD, ASD	
Spinal cord (rat)	1:6	[128]	Female bias:—	[110]
			Male bias: PD	
Cerebellum (mouse) Cerebellum (human)	1:1 1:4	[117] [115]	Female bias:—	[110, 114]
			Male bias: PD, ASD	

*AD* Alzheimer’s disease, *PD* Parkinson’s disease, *MD* major depression, *ANX* anxiety disorders, *SZ* schizophrenia, *ASD* autism spectrum disorders

Testosterone’s effects are state dependent. Under low oxidative stress conditions, testosterone and its metabolite 17β-estradiol are neuroprotective via the estrogen receptor. We did not observe protective effects in C6 glial-derived cells. Interestingly, our data showed a window of opportunity for neuroprotection by sex hormones. If neuronal-derived cells were hormone-deficient at least 1 h, neither testosterone nor estrogen protected cells from subsequent oxidative stress insults. These results are consistent with findings from the Women’s Health Initiative concerning the loss of estrogen-mediated protection in menopausal women. Specifically, estrogen was protective in women less than 10 years from menopause [130]. Estrogens are associated with decreased homocysteine, a marker of oxidative stress [131], in women within 10 years from menopause [130, 132]. However, homocysteine levels greater than 8 umol/L in postmenopausal women were associated with negative effects of estrogen [133]. Further, homocysteine levels greater than 14 umol/L in individuals over 60 years of age were linked with Alzheimer’s disease risk [134, 135], which is more prevalent in postmenopausal women [11]. Since our results show that estrogens were not protective in an oxidative stress environment, homocysteine levels may be useful as a biomarker for the “window of opportunity” for estrogen protection.

The classical cytosolic androgen receptor did not mediate androgen’s effects on neuroprotection, nor did classical androgen receptor antagonists affect androgen-mediated toxicity. Based on these results, medical use of androgen receptor antagonists is unlikely to interfere with androgen’s neuroprotective or damaging effects in neuronal and glial cells. Currently, androgen receptor antagonists are used to treat benign prostatic hyperplasia, prostate cancer, alopecia, hypersexuality, precocious puberty, and transgender transition in men, whereas in women, these drugs are used to treat acne, hirsutism, hyperandrogenism, and amenorrhea.

In contrast, the use of estrogen receptor antagonists could have a significant adverse effect by blocking testosterone- and 17β-estradiol-mediated neuroprotection. Currently, estrogen receptor antagonists are used in men and women to treat multiple conditions. Estrogen receptor antagonists are used to treat gynecomastia, breast cancer, and hypogonadism in men; breast cancer, ovulation induction, and transgender transition in women. Notably, the use of estrogen receptor antagonists (i.e., tamoxifen) are associated with increased Parkinson’s disease risk in women [136–138]. However, the role of estrogen receptor antagonists in Alzheimer’s disease risk in women is less clear [139–143]. No studies have examined the impact of estrogen receptor antagonists on CNS conditions in men.

## Perspectives and significance

The effects of androgens and estrogens on neuronal and glial cell viability are dependent on the cellular environment. In healthy neuronal cells, androgens and estrogens are protective against oxidative stress insults via the estrogen receptor. However, in unhealthy (e.g., high oxidative stress) neuronal and glial cells, sex hormones have negative effects on cell viability by exacerbating oxidative stress-induced cell loss. Additionally, the non-genomic AR45 receptor is involved in androgen’s damaging effects, but it is unknown which receptor mediates estrogen’s damaging effects. These state-dependent effects of sex hormones may mediate the observed sex differences in oxidative stress-associated CNS disorders.

## Data Availability

The datasets used and/or analyzed during the current study are available from the corresponding author upon reasonable request.
